# Neuronal densities and vascular pathology in the hippocampal formation in CADASIL

**DOI:** 10.1016/j.neurobiolaging.2020.09.016

**Published:** 2021-01

**Authors:** Yumi Yamamoto, Yoshiki Hase, Masafumi Ihara, Ahmad Khundakar, Sigrun. Roeber, Marco Duering, Raj N. Kalaria

**Affiliations:** a Translational and Clinical Research Institute, Newcastle University, Campus for Ageing and Vitality, Newcastle upon Tyne, UK; b Department of Molecular Innovation in Lipidemiology, Suita, Osaka, Japan; c Department of Neurology, National Cerebral and Cardiovascular Center, Suita, Osaka, Japan; d School of Health and Life Sciences, Teesside University, Middlesbrough, Tees Valley, UK; e Institut für Neuropathologie, LMU München; f Institute for Stroke and Dementia Research (ISD), University Hospital, LMU Munich & Munich Cluster for Systems Neurology (SyNergy), Munich, Germany

**Keywords:** CADASIL, Cognitive impairment, Hippocampus, Neuronal density, Vascular dementia white matter damage

## Abstract

Cerebral autosomal dominant arteriopathy with subcortical infarcts and leukoencephalopathy (CADASIL) is the most common form of hereditary cerebral small vessel disease. Previous neuroimaging studies have suggested loss of hippocampal volume is a pathway for cognitive impairment in CADASIL. We used unbiased stereological methods to estimate SMI32-positive and total numbers and volumes of neurons in the hippocampal formation of 12 patients with CADASIL and similar age controls (young controls) and older controls. We found densities of SMI32-positive neurons in the entorhinal cortex, layer V, and cornu ammonis CA2 regions were reduced by 26%–50% in patients with CADASIL compared with young controls (*p* < 0.01), with a decreasing trend observed in older controls in the order of young controls> older controls ≥ CADASIL. These changes were not explained by any hippocampal infarct or vascular pathology or glial changes. Our results suggest notable loss of subsets of projection neurons within the hippocampal formation that may contribute to certain memory deficits in CADASIL, which is purely a vascular disease. It is likely that the severe arteriopathy leads to white matter damage which disconnects cortico-cortical and subcortical-cortical networks including the hippocampal formation.

## Highlights

• Hippocampal volume loss was associated with cognitive dysfunction in CADASIL.
• SMI32+ neurons in entorhinal cortex (V) and CA2 regions were reduced in CADASIL.
• Hippocampal cellular changes were not explained by any infarct pathology.
• Neuronal volumes or glial cell numbers per neuron were not changed.
• Selective loss of projection neurons within the hippocampal formation in CADASIL.

## 1 Introduction

Loss of neurons and subsequent atrophy of the hippocampal formation within the cornu ammonis (CA) fields, subiculum and entorhinal cortex (EC), are common pathological changes observed in different stages of cognitive impairment including mild cognitive impairment (Pennanen et al., 2004), Alzheimer's disease (AD) (Gómez-Isla et al., 1996; Kril et al., 2002a), and dementia with Lewy body (Harding et al., 2002). In the EC, layer II appears to be the first affected area, which is the origin of input signals to the hippocampal CA fields through the perforant path, whereas EC layer V (ECV) receives major hippocampal output from the CA1 and returns the signal to the neocortex and subcortical structures such as the thalamus. It is thought that layers II and V in the EC play critical roles in memory processing (Knierim, 2015).

Previous studies including ours (Gemmell et al., 2012; Kril et al., 2002b; Zarow et al., 2005) quantified densities of hippocampal neurons in vascular dementia (VaD) with the overall findings of decreases in numbers or volumes of CA1 and CA2 pyramidal neurons (Kril et al., 2002b; Zarow et al., 2005). However, there were contradictory findings in these studies that are likely explained by the diverse underlying pathology and inconsistent definitions associated with VaD (Kalaria, 2016; Kalaria et al., 2004).

Cerebral autosomal dominant arteriopathy with subcortical infarcts and leukoencephalopathy (CADASIL) is the most common form of hereditary small vessel disease (SVD) caused by mutations in the *NOTCH3* gene (Chabriat et al., 2009). Patients with CADASIL develop severe arteriopathy and subcortical ischemic infarcts, which often lead to the manifestation of migraine with aura, subcortical infarcts, mood disturbances, and eventually cognitive impairment (Chabriat et al., 2009). The profile of cognitive impairment in CADASIL is similar to that in sporadic VaD and manifests as impairments in processing speed and executive function in contrast to that in AD, that is, word recall and recognition memory but relatively preserved semantic fluency (Buffon et al., 2006; Levy and Chelune, 2007). In the late stages, patients with CADASIL develop variable degrees of vascular cognitive impairment (VCI) (Hachinski et al., 2006; Skrobot et al., 2018). However, the impact of the vascular pathology on neurons and thus on cognitive function domains including memory in patients with CADASIL remains to be fully determined.

To our knowledge, previous studies have not systematically investigated neuronal density changes in patients with CADASIL. Because of the genetically defined disease and younger age of onset, alterations in CADASIL are of purely vascular origin and brains are predominantly free of any Alzheimer type of pathology. However, a single case described as a cortical form of CADASIL with cerebral Aβamyloidosis exhibited classical subcortical vascular pathology with numerous lacunar infarcts (Paquet et al., 2010). Cerebral atrophy underlying cognitive dysfunction in CADASIL appears relatively uniform although the anterior temporal poles are often severely affected (Yamamoto et al., 2009) that may extend to other regions of the temporal lobe. Consistent with this, previous comprehensive neuroimaging studies in a large cohort indicated that hippocampal volume was an independent predictor of cognitive function in CADASIL (O'Sullivan et al., 2009). Hippocampal volumes were reduced in patients with CADASIL who developed dementia and in a subgroup progressing to dementia before age 60 years. On the basis that hippocampal atrophy is an important pathway of cognitive impairment also in vascular disease (Kalaria and Ihara, 2017), we reasoned that an examination of hippocampal neurons and associated axonal changes in the temporal stem in patients with CADASIL using specific markers such as SMI32 (Gottron et al., 1995; Lindner et al., 2009) may provide insights into the neuronal substrates of cognitive impairment in VaD (Gemmell et al., 2012). SMI32, which comprises nonphosphorylated neurofilaments-M and -H, is found in pyramidal projection neurons (type I), and its depletion is a sensitive marker of chronic ischemic stress (Leifer and Kowall, 1993; Voelker et al., 2004), but SMI32(+) neurons have also been reported in AD and dementia with Lewy body (Thangavel et al., 2009). Although SMI32 abundantly exists in neuronal cell bodies and dendrites, it is absent in normal myelinated axons unless they are damaged and dysmyelinated (Trapp et al., 1988). SMI32 staining, which appears as continuous lines, small dots, or ovoids (retraction bulbs), is detected in axons only after a longer dysmyelination periods, and thus, it is a useful indicator of chronic axonal damage and possibly axonal degeneration (Lindner et al., 2009).

## 2 Materials and methods

### 2.1 Subjects and cases

Table 1 provides the demographic details of each group used to quantify vascular pathology, neuronal densities, and volumes in CADASIL (n = 12), young controls (n = 10), and older controls (n = 7). Brain tissue was obtained from 4 resources: the Newcastle Brain Tissue Resource, Newcastle General Hospital; MRC London Brain Bank for Neurodegenerative Diseases; the MRC Sudden Death Brain and Tissue Bank (Millar et al., 2007), the University of Edinburgh; and the University Hospital at LMU Munich, Germany. CADASIL diagnosis was confirmed based on the presence of *NOTCH3* gene mutations and associated clinical features. All patients with CADASIL were cognitively impaired at death. Review of available notes on patients with CADASIL indicated that they at least met the mild VCI classification (Skrobot et al., 2018) Use of brain tissue was approved by the local research ethics committee of the Newcastle upon Tyne Hospitals NHS Foundation Trust, the Newcastle Brain Tissue Resource committee, and the ethics committees overseeing the brain banks at the other respective sites.

**Table 1:** Demographic details of patients with CADASIL and controls

Group(n)	Age (yr)	Sex	Mutation	Duration (yr)	Notable clinical features and risk factors^a^
CAD1	44	F	Arg153Cys	8	Cardiac arrhythmias; mild VCI
CAD2	53	F	Arg133Cys	6	No vascular risk; mild VCI
CAD3	55	M	Arg558Cys	11	Brief history of gout; mild VCI
CAD4	58	M	Arg985Cys	13	No vascular risk; severe VCI or dementia
CAD5	59	M	Arg169Cys	12	No vascular risk; severe VCI or dementia
CAD6	61	M	Arg169Cys	10	Obesity (~55 y); severe VCI or dementia
CAD7	66	F	D239_D253del	23	No vascular risk, obesity; severe VCI or dementia
CAD8	68	F	Arg133Cys	18	Smoking history; severe VCI or dementia
CAD9	68	M	Arg153Cys	28	Smoking, prostate tumor; severe VCI or dementia
CAD10	63	M	Arg141Cys	10	Enlarged thyroid; no vascular risk; mild VCI
CAD11	65	M	Arg141Cys	13	Parenchymatous goiter; no vascular risk; mild VCI
CAD12	52	M	Arg141Cys	10	No vascular risk; severe VCI or dementia
CADASIL group (mean) (12)	59	8M/4F	75% mutations in exon 4	12	Predominant characteristics of CADASIL syndrome, majority with no vascular risk factors
Young controls (10)	54	3M/7F	-	-	No cerebrovascular or neurodegenerative disorders
Older controls (7)	79	3M/4F	-	-	

Mean age (y) is shown for CADASIL (CAD), similar age of young controls and older controls. There were no statistical differences in mean ages between the patients with CADASIL and control participants (*p* > 0.05). In most patients with CADASIL, there was a cystine (Cys) replacement.

Key: CADASIL, cerebral autosomal dominant arteriopathy with subcortical infarcts and leukoencephalopathy; VaD, vascular dementia; VCI, vascular cognitive impairment.

a Mild VCI and severe dementia or VaD were defined as per VICCCS criteria (Skrobot et al., 2018) from available case notes.

### 2.2 Histopathology and vascular pathology scores

**Fig. 1:**
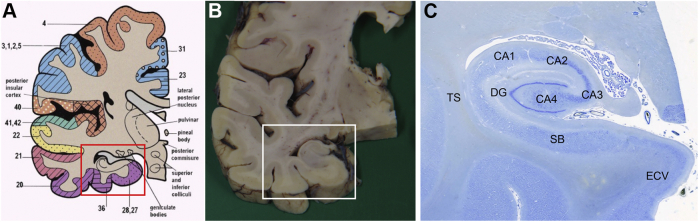
(A) Diagram of the coronal block from one hemisphere of the cerebrum at the level of the anterior hippocampal formation; (B) Coronal fixed tissue slice from the cerebrum of a 79-year-old control participant at the level of the hippocampal formation taken for neuropathological examination; (C) Section stained with Nissl showing the complete hippocampal formation. (A, B) Boxes show delineated sample areas and (C) shows the anatomical regions of the temporal stem (TS), the entorhinal cortex layer V (ECV), and cornu ammonis (CA1 and CA2), which were assessed for pathological quantification. Abbreviations: DG, dentate gyrus; SB, subiculum.

As previous imaging studies had suggested changes in the medial temporal lobe are associated with some memory deficits in patients with CADASIL, we concentrated on the hippocampal formation (Table 2). All the brain tissues were collected from the other centers and analyzed in Newcastle, preventing any sampling bias or introducing variation in the analysis. Temporal lobe blocks at the coronal level of the lateral geniculate nucleus were obtained for assessment of neuronal density. Cerebrovascular lesions including SVD pathology were assessed as described previously (Deramecourt et al., 2012). Sclerotic index was determined as described previously (Craggs et al., 2012). Axonal changes in the stem of the temporal lobe or temporal stem were assessed at the level of the anterior hippocampus in adjacent sections used to determine neuronal densities (Fig. 1). For neuronal counts and volumes, formalin-fixed, paraffin-embedded blocks were serially sectioned at thicknesses of 30 μm. They were immunostained using mouse anti–nonphosphorylated neurofilament H (anti-SMI32, Covance, CA; 1:500) monoclonal antibody to identify large pyramidal neurons and damaged axons in the white matter (WM) (Lindner et al., 2009; Voelker et al., 2004) and then counterstained with 0.1% cresyl fast violet to reveal the total neuron population.

**Table 2:** Spectrum of hippocampal pathology in CADASIL cases^a^

Group(n)	Mutation	Vascular path scores^a^	Arteriolosclerosis (0–3)	PVS (0–3)	Microinfarcts Y/N	CA1 atrophy (0–3)	Other features
CAD1	Arg153Cys	6	1	1	N	0	Normal size of hippocampi
CAD2	Arg133Cys	8	2	1	N	1	Gliosis
CAD3	Arg558Cys	10	2	2	Y	2	Gliosis
CAD4	Arg985Cys	7	2	2	N	1	Gliosis
CAD5	Arg169Cys	8	3	2	N	1	Gliosis; relatively preserved
CAD6	Arg169Cys	6	3	3	Y	2	b,eIntimal thickening in vessels
CAD7	D239_D253del	9	3	2	Y	2	Gliosis; some vessels with thrombotic changes
CAD8	Arg133Cys	7	3	3	Y	2	bcArteriosclerotic thickening
CAD9	Arg153Cys	9	3	3	Y	2	Arteriosclerotic thickening
CAD10	Arg141Cys	7	2	2	Y	2	e Arteriosclerotic thickening
CAD11	Arg141Cys	8	2	2	Y	2	e Arteriosclerotic thickening
CAD12	Arg141Cys	8	3	3	Y	3	e Arteriosclerotic thickening
CADASIL group (mean)	No clear correlation	7.8	2	2	Y	2	Overall gliosis and hippocampi relatively preserved from direct ischemic lesions
Ycont (10)^d^	-	3	1	N	N	0	No cerebrovascular or neurodegenerative disorders
Ocont (7)^d^	-	5.5	1	N	N	1	

Data show extent of hippocampal pathology (scores of 0–3; 0 = none, 1 = mild, 2 = moderate, 3 = severe).

Key: CADASIL, cerebral autosomal dominant arteriopathy with subcortical infarcts and leukoencephalopathy; PVS, periventricular space.

a Scores were calculated as previously described (Deramecourt et al., 2012) out of 10 and the VCING Criteria (Skrobot et al., 2016).

b Evidence of hemosiderin.

c Diffuse amyloid-β plaques were found.

d Braak staging in CADASIL cases and controls were in range 0–II, except 81-year-old control (Supplementary Table S1). In addition, the overall ABC scores were determined to be none to low for the CADASIL cases and all controls (Montine et al., 2012).

e Evidence of general atheromatous disease and occlusion in regions of the cerebrum other than the hippocampal formation of these cases.

To assess neurodegenerative pathology, sections were stained with hematoxylin and eosin for structural integrity and infarcts, Nissl and Luxol fast blue staining for cellular patterns and myelin loss, and Bielschowsky's silver impregnation and Gallyas stain for neuritic pathology. Amyloid-β and tau immunohistochemistry were performed to determine neuritic plaques and Braak staging of neurofibrillary tangles and rate ABC for amyloid β deposits (A), staging of neurofibrillary tangles (B), and scoring of neuritic plaques (C) scores as per National Institute on Aging–Alzheimer's Association guidelines (Table 2). Unless otherwise stated, all the tissues were processed for fixation in 10% buffered formalin and embedded in paraffin following standard protocols established in clinical histopathology services.

### 2.3 Unbiased 3-dimensional stereological quantification

**Fig. 2:**
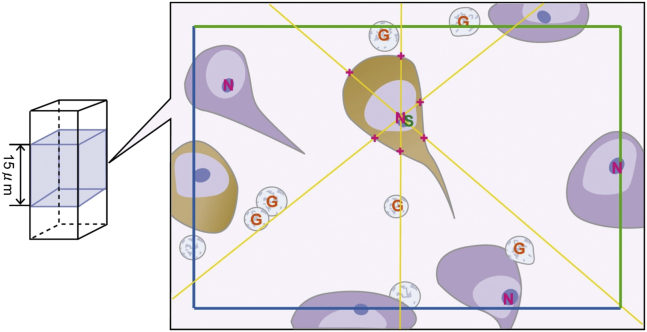
The image shows how 3-dimensional stereology was used to quantify neuronal numbers and volumes. Neurons with nucleolus within counting frame or touching green lines were measured. The middle 15-μm depth was scanned through to identify neurons and glial cells within the dissector box. Glial cells and neurons with a nucleolus inside or touching the green lines were counted and measured for volume estimation. Neurons were marked with N, SMI32-positive neurons (colored brown) with S, and glial cells with G. The intersections of lines with cell membrane were marked by the researcher for volume estimation.

Three-dimensional stereological quantification of cell density and volume was determined essentially as described previously (Gemmell et al., 2012, 2014). We also ensured sections from the hippocampal formation from both CADASIL and controls were free of any direct tissue damage due to infarction. Thus, purely effects of remote ischemic injury were evaluated on the hippocampal formation. Briefly, 2 randomly selected 30-μm sections per sample were analyzed using a Visiopharm Integrator System (Visiopharm, Denmark). The precise regions of interest (ROI) in the EC (layer V, ECV) and hippocampus (*cornu ammonis,* CA1 and CA2; Fig. 1) were defined as previously described (Amaral and Insausti, 1990). Cell counts and volume estimations were conducted using a 100x oil-immersion objective on a Zeiss Axioplan Photomicroscope, whereas the XY and Z motorized stage (Prior ProScan II, Prior Scientific Instruments Ltd, UK) provided systematic random sampling. The unbiased dissector and nucleator method were used to estimate density and size of neurons and glial cells (Fig. 2). Tissue section thickness was measured using a Heidenhain gauge (Heidenhain GB Ltd, UK) at every 10th field during analysis. Approximately 40–80 fields per ROI were analyzed to achieve an identical sampling fraction for each ROI. Neurons were counted only when their nucleoli could be identified, and the cell body was either fully inside the dissector box measuring 56.43 × 45.14 μm or touching 1 of the 3 nonforbidden planes (right, top, and upper side) within the dissector height, which was set at 15 μm. SMI32-positive neurons were counted separately from the total neuron population.

### 2.4 SMI32-positive axons

Previous studies have shown that chronically damaged axons show accumulation of nonphosphorylated neurofilaments H which is revealed with SMI32 immunoreactivity (Lindner et al., 2009). As described previously (Craggs et al., 2014), SMI32-stained sections from CADASIL and young and older controls were viewed under an Olympus BX51 upright microscope coupled with an mbf Bioscience CX9000 camera and analyzed with a Stereo Investigator (MBF Bioscience, VT, USA) using the Space Balls probe. SMI32 axons were examined in the whole temporal stem WM region as the ROI (Fig. 1).

### 2.5 Statistical analyses

Comparisons between CADASIL, young control, and old control participants were made using Kruskal-Wallis and Mann-Whitney U tests incorporated in SPSS, version 17.0. Correlations between a number of variables including neuronal densities as actual values, SMI32 positive and negative stained axonal densities determined as percent area, and MMSE scores were tested using Spearman's rank order correlation.

## 3 Results

### 3.1 Neuronal and vascular pathology in the hippocampal formation

Routine neuropathological examination of sections from the medial temporal lobe of CADASIL cases was unremarkable. They did not show marked atrophy compared with those from young or older controls. Hippocampi were largely of the expected size (Table 2). We noted mild to moderate hippocampal sclerosis in CA1 region in CADASIL cases. Immunostaining with antibodies to pathological markers of AD confirmed the general absence of any significant pathology or neurofibrillary tangle burden both in CADASIL cases and all controls (Table 2; Supplementary Table S1), where available Braak staging for neurofibrillary tangles was recorded to be in range 0–II in all but one old control participant, who exhibited Braak stage III. The overall ABC scoring for AD type of pathology was also determined to be none to low in the CADASIL cases and controls (Montine et al., 2012).

**Fig. 3:**
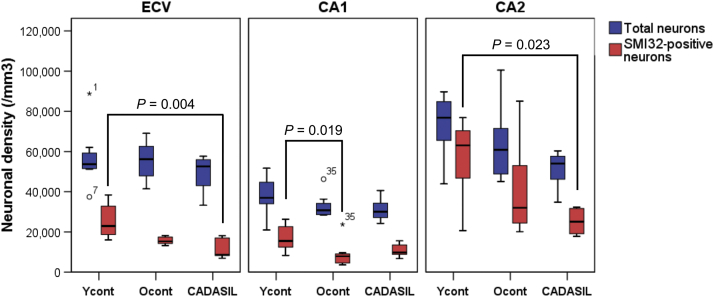
Neuron densities in patients with CADASIL and control participants. Differences in SMI32-positive and total cell densities in patients with CADASIL in the entorhinal cortex, layer V (ECV) and hippocampus regions CA1 and CA2 relative to young and older controls. SMI32-positive neuronal density was decreased in CADASIL and older controls (B). ∗: Significance (*p* < 0.05) and +: trend (*p* < 0.1) versus age-matched controls or between controls. Error bars ± 2SE. Abbreviations: CADASIL, cerebral autosomal dominant arteriopathy with subcortical infarcts and leukoencephalopathy.

To determine if neuronal status was influenced by vascular lesions or infarctions in the hippocampal formation of patients with CADASIL, we also determined several features of microvascular pathology. We found that the mean vascular pathology scores incorporating cortical and subcortical areas (Deramecourt et al., 2012) in patients with CADASIL were greater than those in older controls (Table 2). Although we noted variable arteriopathy with a median score of 2, except for a couple of cases, microvascular pathology in the hippocampal formation was not remarkable in CADASIL. There were no obvious small or large infarcts in any of the cases or controls. We found that the sclerotic index of hippocampal in relation to medial temporal lobe vessels in patients with CADASIL was significantly greater than that in young controls (0.43 vs. 0.30, *p* < 0.001). We found no clear relationship between hippocampal neuronal or microvascular pathology and any particular *NOTCH3* genotype. However, the most severe vascular pathology was present in the participant with a *NOTCH3* mutation in exon 11. Overall, the lack of infarctions or any neuronal sclerosis in CA1 region of the hippocampal formation could not explain the selective neuronal changes we observed (Fig. 3).

### 3.2 Pyramidal neuronal densities and volumes

Relative to total neurons detected with conventional tinctorial staining, we had a particular interest in the status of SMI32-positive neurons. We first noted that proportional densities of SMI32-positive neurons to total neurons in young control participants were 44.5% in the ECV, 45.4% in CA1, and 75.9% in CA2 regions (Fig. 3). In patients with CADASIL, percentage of SMI32-positive neurons was dramatically lower by 25.5% and 49.5% in the in the ECV and CA2 regions (*p* = 0.008 and *p* = 0.037), respectively. There were also fewer SMI32-positive neurons in the CA1, but these were not significantly different from young control participants at the 5% significance level. Compared with the young controls, we also found SMI32-positive neurons to be lower in old control participants, but differences in mean densities and percentage of SMI32-positive neurons were significant only in the CA1 (*p* = 0.019 and *p* = 0.037) and only trend was observed in ECV (*p* = 0.075 and *p* = 0.094).

After these observations, correlation analysis between SMI32-positive and -negative neuron densities was performed to determine whether the observed decrease in the SMI32-positive neurons in old control participants and patients with CADASIL was a true indication of large pyramidal neuronal loss or merely reflecting loss of SMI32 immunoreactivity. As expected, controls showed correlation between SMI32-positive and SMI32-negative neuronal density (young and old controls combined; CA1: R = −0.471, *p* = 0.076; CA2: R = −0.675, *p* = 0.006). However, there was no significant correlation in any of the regions in CADASIL cases. These results indicated that while only the expression of SMI32 was reduced in older controls, small numbers of large pyramidal neurons were lost in the hippocampus of CADASIL (coincident with decreased SMI32 expression).

We found the ECV and CA2 regions had the largest estimates of neurons in range 70,000–80,000 per mm^3^. In contrast to SMI32-positive neurons, total neuronal densities were relatively preserved in the in the ECV and CA1 regions in CADASIL compared with young control and old control groups but tended to be reduced in the CA2 region (*p* = 0.071, Kruskal-Wallis test) (Fig. 3B).

The mean volumes of neurons in young control in the ECV, CA1, and CA2 regions were 1788 μm3, 2303 μm3, and 2765 μm3, respectively. There were no detectable differences in neuronal volumes between CADASIL and young control participants or between young control and old control groups (*p* > 0.05).

The density of glial cell was similar between groups in any of the regions (*p* > 0.05). A difference was only found in the glial cell number per neuron between young and old controls (*p* = 0.042) in CA1, although it may be the result of the decreased neurons and retained glial populations.

### 3.3 Axons in the temporal stem

**Fig. 4:**
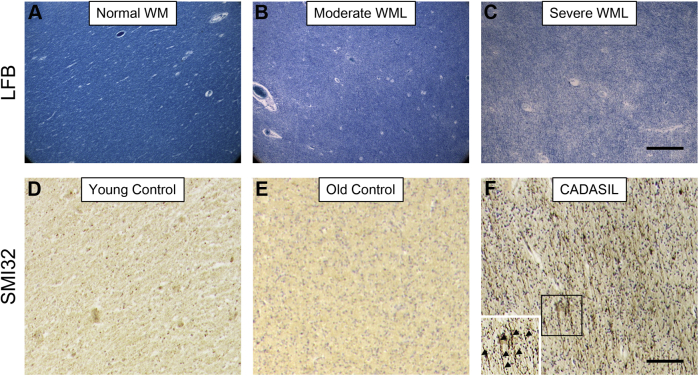
Axonal abnormalities in the temporal stem of patients with CADASIL. Examples of myelin (LFB) staining and SMI32 immunostaining show severity of demyelination and axonal changes in the temporal stem WM of (A, D), young controls; (B, E), older controls; and (C, F) patients with CADASIL. Highly contrasting SMI32-immunostained structures were evident in CADASIL (inset in F). Bar = 100 μm. Abbreviations: CADASIL, cerebral autosomal dominant arteriopathy with subcortical infarcts and leukoencephalopathy; WM, white matter.

Consistent with our previous report (Craggs et al., 2014), we found intense and abundant SMI32 staining in the temporal stem WM of patients with CADASIL compared with all controls (Fig. 4). Varying degrees of SMI32-positive axonal degeneration, such as fragments of axons, swollen segments, and ‘retraction bulbs,’ were evident in the temporal stem of all CADASIL cases, whereas not in young controls. There was a general absence of SMI32-positive changes in control samples in contrast to the CADASIL samples. We did not undertake further quantitative analyses of this more narrowed ROI given the length density of SMI32-positive axons was doubled in patients with CADASIL compared with young controls as reported previously (Craggs et al., 2014).

## 4 Discussion

Our study provides the first quantitative data on selective neuronal changes underlying cognitive dysfunction, specifically in the medial temporal lobe–related memory deficits in CADASIL. Our observations are substantiated by the fact that densities of hippocampal neurons and damaged axons were quantified using current state-of-the-art unbiased stereological techniques. Although the total neuronal densities in the hippocampal formation of patients with CADASIL were relatively preserved, there were marked losses of ~26%–50% in SMI32-positive neurons in the ECV and CA2 regions. Another remarkable observation was the differential loss of neuronal densities with age in that the older control group had fewer total neurons than young controls in the ECV. Specific losses in pyramidal neurons of the ECV are particularly of note because this region has been implicated in early phases of neurodegenerative disorders such as AD. It has been suggested that thread-like neuronal processes and tau neurites develop first in layer II and then spread via axons to layer V of the EC. Losses in SMI32 neurons in both the ECV and CA2 can only be explained by a vascular etiology rather than any protein inclusions and propagation or transmission as suggested to take place in AD (Braak and Del Tredici, 2015; Gibbons et al., 2019; Goedert et al., 2017). The ECV receives inputs intrinsically from EC layers II and III, which connect the CA regions of the hippocampus via the performant pathway and the CA1 region then closes the loop with medial and lateral projections to the ECV via the subiculum with a subsequent outflow to the thalamus and isocortical regions (Knierim, 2015; Ohara et al., 2018). Any memory deficits associated with CADASIL likely result from the weakening of this intrinsic hippocampal-entorhinal circuitry. It should be emphasized that these specific neuronal changes occur in the general absence of any type of Alzheimer pathology either in the EC or hippocampus proper and are purely driven by processes associated with SVD.

We have previously shown that the CA1 region of the hippocampus is particularly vulnerable, irrespective of the presence of AD type of pathology in poststroke survivors who develop VCI or dementia (Gemmell et al., 2012, 2014). We previously suggested there is a strong vascular basis of neuronal density and volume reductions in the CA1 and CA2 regions, respectively. CADASIL is characterized by executive dysfunction with relatively preserved hippocampal functions (e.g., episodic memory) until progression into late stages (Buffon et al., 2006; O'Sullivan et al., 2009). The lack of total neuronal density changes in the hippocampus of patients with CADASIL suggests there is less extensive involvement of the entire hippocampal structure even though these patients are clinically impaired and develop VCI.

Previous studies have shown that SMI32 immunoreactivity is depleted after cerebral ischemia, especially in chronic lesions (Leifer and Kowall, 1993). Our observations suggest that patients with CADASIL likely experienced chronic ischemic stress causing almost similar loss of SMI32-positive neurons in hippocampus and EC as in older controls. A few studies reported neuronal hypertrophy in AD and mild cognitive impairment, implying that it is involved in their pathology (Iacono et al., 2008). However, there were no significant differences in neuronal volumes although neuronal volumes in CA2 regions tended to be greater compared with younger controls. Our results are supported by the observations of Viswanathan et al. (2006) and Mesulam et al. (Mesulam et al., 2003), which noted lack of significant changes in apoptotic neuron number and cholinergic denervation, respectively, in the hippocampus of patients with CADASIL. We further investigated the profile of mild neuronal loss in the hippocampal formation and found selective vulnerability of SMI32-positive neurons. SMI32, a 200 kDa nonphosphorylated neurofilament, is expressed in large pyramidal projecting neurons, which are most abundantly found in the long association pathway connecting frontal, temporal, and parietal cortices (Hof et al., 1995). Our results not only confirm the previously reported vulnerability of these pyramidal neurons to ischemic stress (Leifer and Kowall, 1993; Thangavel et al., 2008, 2009) but also suggest that WM disconnection precedes neuronal loss in CADASIL. It is not unlikely that microstructural damage in WM tracts connecting infarcted regions with distant cortex causes secondary cortical neurodegeneration as a mechanism for brain atrophy (Duering et al., 2012, 2015). It is also plausible that the SVD type of changes in subcortical structures cause a chronic hypoxic state (Fernando et al., 2006; O'Sullivan et al., 2005), which makes the WM more vulnerable with increasing age (Ihara et al., 2010).

We also found profound abnormal SMI32 accumulation in the temporal stem WM of patients with CADASIL. This suggests chronic axonal damage (Lindner et al., 2009) secondary to the widespread demyelination in subcortical WM in CADASIL. The stem of the temporal lobe (temporal stem) forms a bridge between the temporal lobe and other regions of the brain and is traversed by the uncinate fasciculus, inferior occipitofrontal fasciculus, and Meyer's loop of the optic radiation (Kier et al., 2004). The uncinate fasciculus is a monosynaptic corticocortical route of interaction between the temporal and frontal lobes. Reduction in volume or axonal abnormalities of the temporal stem would affect both efferent and afferent WM tracts as they enter and exit the temporal lobe. Increased water diffusion likely results from disruption and loss of axonal membranes and myelin stem that has also been described in patients with AD (Hanyu et al., 1998).

The disconnection syndrome is a theory that brain dysfunction can be, at least in part, attributed to disrupted associative connections between various brain regions by WM abnormality (Catani and Ffytche, 2005). In AD, for example, the functional anterior-posterior disconnection and changes in medial temporal structures were associated with delayed memory (Grady et al., 2001). Patients with CADASIL exhibit rather specific spatial distribution of WM lesions as shown by magnetic resonance imaging (Coulthard et al., 2000) and our previous clinicopathological studies (Craggs et al., 2014). Prior studies had further shown that *NOTCH3* mutation carriers display WM abnormalities early in the disease that is detectable well before the occurrence of stroke, cognitive impairment, and disability (Chabriat et al., 1998). Consistent with this, neuronal apoptosis may contribute to cortical atrophy and cognitive impairment in patients with CADASIL and that may result from axonal damage in the underlying WM (Gray et al., 2007). Moreover, experimental studies in *Notch3*^*R169C*^ mice suggest that segmental intramyelinic edema is an early conspicuous WM change in CADASIL (Cognat et al., 2014). Thus, it is plausible that disrupted frontal lobe connectivity with the temporal lobe underlies the cognitive deficits in CADASIL. Notwithstanding, Tatsch et al. (2003) considered disconnections by subcortical lesions as one of the causes of reduced cortical metabolism and cognitive dysfunction in CADASIL.

We used CADASIL brain tissues from several different resources, which inevitably could result in the inconsistency in diagnostic criteria, neuroimaging methods, and neuropsychological tests. The limitations of the present study included the lack of a full spectrum of neuropsychometric scores and quantitative magnetic resonance imaging data for all CADASIL cases, which prevented us from properly relating the pathological changes to temporal lobe neuroimaging and cognitive function data. Establishing large prospective studies of patients with CADASIL by collaborating centers would facilitate studies into the etiology of medial temporal lobe atrophy in neurodegenerative diseases such as AD and CADASIL as a model of sporadic SVD leading to dementia.

## 5 Conclusions

Our results show selective loss of neurons in the hippocampal formation, suggesting this is a proxy for the observed hippocampal volume loss in CADASIL as demonstrated by previous neuroimaging studies (O'Sullivan et al., 2009). Our results also suggest that WM integrity in the temporal stem is compromised and is a factor in the observed cell loss in the hippocampal formation, particularly in the ECV. These observations collectively may explain the cognitive dysfunction associated with the temporal lobe in CADASIL. In future investigations, it is imperative to evaluate neuronal densities in other areas of the temporal lobe similar to the frontal lobes (Bussière et al., 2003; Foster et al., 2014; Hof et al., 1990).

## Disclosure statement

None related to this manuscript.

## CRediT authorship contribution statement

**Yumi Yamamoto:** Funding acquisition, Formal analysis, Data curation, Writing - review & editing. **Yoshiki Hase:** Formal analysis, Funding acquisition, Writing - review & editing. **Masafumi Ihara:** Writing - review & editing, Data curation. **Ahmad Khundakar:** Writing - review & editing, Data curation. **Sigrun. Roeber:** Writing - review & editing. **Marco Duering:** Writing - review & editing, Data curation. **Raj N. Kalaria:** Conceptualization, Methodology, Validation, Formal analysis, Writing - review & editing, Writing - original draft, Supervision, Project administration, Funding acquisition.

## Appendix A Supplementary data

Supplementary Table S1

Data Profile
