# Supplementary material for: Neuronal densities and vascular pathology in the hippocampal formation in CADASIL
Source: Neurobiol Aging. 2021 Jan;97:33–40. doi: 10.1016/j.neurobiolaging.2020.09.016 (PMC7758782; doi:10.1016/j.neurobiolaging.2020.09.016)
Supplement: Supplementary Table S1 [file mmc1.docx]

**Supplementary Table S1: Demographic Details of and Hippocampal Features in Young and Old controls**

| **Group (n)** | **Age (yr)** | **Sex** | **VCI and Pathological Features*** |
| --- | --- | --- | --- |
| **Young Controls (10)** | | | |
| 1 | 41 | F | Sudden death, no significant pathology, Braak 0 |
| 2 | 49 | F | No impairment, no significant pathology, Braak 0 |
| 3 | 51 | M | Sudden death, no significant pathology, Braak 0 |
| 4 | 51 | F | No impairment, no significant pathology, Braak n/a |
| 5 | 53 | M | No impairment, no significant pathology, Braak n/a |
| 6 | 54 | F | No impairment, no significant pathology, Braak 0 |
| 7 | 55 | M | No impairment, no significant pathology, Braak n/a |
| 8 | 58 | F | No impairment, no significant pathology, Braak0 |
| 9 | 59 | F | No impairment, no significant pathology, Braak n/a |
| 10 | 64 | F | No dementia, no significant pathology, Braak 0 |
| **Old Controls (7)** | | | |
| 1 | 78 | M | No dementia, age-related changes, Braak II |
| 2 | 79 | M | No dementia, no significant pathology, Braak 0 |
| 3 | 74 | F | No dementia, no significant pathology, Braak 0 |
| 4 | 78 | F | No dementia, no significant pathology, Braak 0 |
| 5 | 81 | M | No dementia, age-related changes, Braak III |
| 6 | 89 | F | No dementia, age-related changes, Braak II |
| 7 | 81 | F | No dementia, age-related changes, Braak I |

Table shows distribution of young and older controls from which brain tissues were used in the study. Controls, mean age (54, SD 4.5) was not significantly different to mean age of CADASIL group (mean 58, SD= 7.5; P>0.05; Table 1). *Subjects died of various unrelated causes including carcinoma and medication overdose. No impairment relates to cognition. There was no evidence of significant cerebrovascular or neurodegenerative disease in any of the controls; the ABC scores were found to be A0.5, B.1.2, C.0.5 i.e. none to low. The post-mortem intervals ranged 12-36 hr, which was not different from that of CADASIL cases. Abbreviations: Braak, neurofibrillary pathology staging; n/a, not available; NPD, no pathological diagnosis.
